# Lessons for the Future of NAMs from History, Philosophy and Social Studies of Science

**DOI:** 10.1177/02611929241267763

**Published:** 2024-08-06

**Authors:** Rachel A. Ankeny, Gail F. Davies, Robert G.W. Kirk, Alexandra L. Whittaker, Jane Johnson

**Affiliations:** 1 Philosophy Group, 4508 Wageningen University & Research, Wageningen, The Netherlands; 2 School of Humanities, 1066 The University of Adelaide, Adelaide, SA, Australia; 3 Department of Geography, 3286 University of Exeter, Exeter, UK; 4 Centre for the History of Science, Technology and Medicine, 5292 University of Manchester, Manchester, UK; 5 School of Animal and Veterinary Science, 1066 The University of Adelaide, Adelaide, SA, Australia; 6 Department of Philosophy, 7788 Macquarie University, Sydney, NSW, Australia

**Keywords:** animal alternatives, animal models, NAMs, New Approach Methodologies, standards, scientific change, social studies of science

## Abstract

This paper explores what we can learn from the humanities and social sciences about how standards operate in and around science, in order to understand more about how ‘the gold standard’ can be shifted away from the use of animals in research and testing, and toward New Approach Methodologies (NAMs). These fields allow us to consider potential futures of NAMs as alternatives, replacements, or complements to animal use in testing and research. As we demonstrate, the questions that we pose and how they are framed are as important as the answers that result. Rather than asking how to ‘redefine the gold standard’, norms and expectations for NAMs must be actively debated and transparently defined. These considerations would be based, in part, on what has been learned in the past from non-human animal models and systems, but also use the norms within the fields from which the NAMs derive in light of the rich broader contexts within which they are being developed. As we argue, notions such as ‘a gold standard’ are limited and must be replaced by contextualised standards that depend on the scientific, sociocultural and other factors that contribute to our understanding of a particular method (new or otherwise) as ‘good’ for a particular purpose.

## Introduction

The rapid development of New Approach Methodologies (NAMs), for use in fields such as regulatory science and drug development and testing, often leads to the promise that such *in vitro*, *in silico* or other non-animal methods can represent the new ‘gold standard’ and reduce reliance on the use of animals in research and testing. The technologies and approaches encompassed by NAMs are broad, yet the central idea of a gold standard in both animal research and its non-animal alternatives is often simply assumed. Work from the humanities and social sciences on how standards operate in and around science in general can be instructive for any project that aims to shift the ‘gold standard’ away from the use of animals in research and testing. Social studies of science can explore how this shift takes place, through studying the frameworks that shape action (such as legal and regulatory documents, standard operating protocols, or value systems), as well as understanding how organisations, individuals and others enact or modify these frameworks in practice. Consequently, there has been an interdisciplinary turn associated with the Three Rs that has resulted in humanities and social science scholars being enlisted to consider questions about *replacement*, *reduction* and *refinement*,^1^ a turn on which this paper builds. As the animal welfare scholar Herwig Grimm and his co-authors state: “The humanities and social sciences are not the fifth wheel: they are as important as natural science and biomedicine in the project of advancing implementation of the 3Rs and developing the 3Rs themselves”.^2^

When reflecting on NAMs and how they might be positioned, justified and understood in relation to non-human animal experimental methods, many initial responses are likely to be grounded in scientific considerations, particularly relating to what counts as evidence for success or equivalency, and what the standards should be for such evidence. However, studies of science — including history, philosophy and geography, together with other socioculturally focused fields, including regulation, law and ethics — can also make important contributions, especially when we wish to understand and drive changes in scientific practices, in part through standard setting. Such fields are well-suited for investigation into the drivers of, and barriers to, change (and stability), how practices emerge in particular contexts and are sustained or evolve over time, and even how purposeful or intentional change can be fostered.

This paper explores what we can learn from these fields about the potential futures of NAMs as alternatives, replacements, or complements to animal use in testing and research. As we demonstrate, the questions that we pose and how they are framed are as important as the answers that result. Thus, rather than asking how to ‘redefine the gold standard’, norms and expectations for various types of NAMs must be actively debated and transparently defined. These considerations should be based, in part, on what has been learned in the past from non-human animal models and systems, but also use the norms within the fields from which the NAMs derive (e.g. stem cell or model organism research) in light of the rich broader contexts within which they are being developed. As we argue, notions such as a ‘gold standard’ are limited and must be replaced by contextualised standards that depend on the scientific, sociocultural and other factors that contribute to our understanding of a particular method (new or otherwise) as ‘good’ for a particular purpose.

## Standards are best understood as sociocultural and material practices

Standards are essential for gauging success or progress in the sciences, but the work and activities associated with establishing standards, and the material possibilities and limitations associated with these activities, are all too often overlooked as secondary to the scientific work that standards and materials make possible.^3^ We tend to focus on the ends created through standards and materials (the transformative knowledge that they make possible), rather than on the means (the standards and materials themselves).

Standards can appear to be boring, but they are far more interesting than they initially appear. Science studies scholars approach standards as ‘sociocultural projects in themselves’ — projects that rely, for their success, on being rendered invisible or taken for granted.^4^ By approaching them as sociocultural projects in themselves, we develop a richer understanding of the conceptual, social and cultural processes involved in establishing and maintaining standards within communities of scientific and regulatory practice over time.

When viewed from a social science perspective, standards are not material ‘things’, but practices that extend over time and space. Standards are generally developed in one context, and then are disseminated to be used in others. Their codification — for example, in law, guidelines and institutional requirements — help standards to ‘travel’ and give them duration and sometimes longevity. However, such travel is not guaranteed, as local contexts differ and practices diverge. Work to align international standards, through what the geographer and social theorist Andrew Barry calls the development of ‘technological zones’, can facilitate collaboration and co-ordination in science through agreed standards of evidence through which scientists make their norms about methodology explicit.^5^ However, these patterns of scientific collaboration are also strongly shaped by government and funder priorities, investment strategies and local research cultures, among many other factors.

Standards around evidence — such as how much and what types of data are relevant, what they are understood to show and whether they are adequate to support arguments about which methods are preferable — may be readily adopted, if they align with state, commercial and scientific interests. Equally, they may be resisted, if signing up to new standards involves the loss, or perceived loss, of influence, assets or control. Ongoing conversations about harmonising regulatory practices around animal research are testament to some of these geographical challenges.^6,7^ These struggles may also be present in attempts to move away from use of animals and animal-derived tests, as in the case of efforts to establish synthetic alternatives for use in the *Limulus* Amoebocyte Lysate (LAL) assay which, in its standard format, uses horseshoe crab blood. A synthetic substitute for horseshoe crab blood was introduced in 2001 and became commercially available in 2003.^8^ It is now an additional test in the European Pharmacopoeia guidance but remains a non-pharmacopeial test in the USA. The validation and uptake of this *replacement* alternative has been limited by this lack of international harmonisation, the market dominance and ready availability of the standard LAL assay, worries about the sourcing and supply of the synthetic alternative, and the lack of regulatory requirements to replace or reduce animal use in this case.^9–11^

These insights clearly illustrate that standards never exist in isolation but must always be viewed in their sociocultural contexts.

## There is no such thing as ‘a gold standard’

A further implication of focusing our attention on the sociocultural domain is that it forces our thinking away from the concept of a *standard* (gold or otherwise) to plural *standards.* The idea of the gold standard has connotations of singularity, of something being the pinnacle of a hierarchy of possible forms of evidence gathering, as it prioritises one form of experimental methodology, the rest of which are inferior by comparison. The idea of a gold standard comes from the economic idea that the standard monetary unit should be based on a fixed quantity of gold. This concept provided a grounding for value, but reduced flexibility, and tended to orient the global economy around one or a small number of nations. The concept was abandoned (and reinstated) at various points in the 20th century, as governments oscillated between managing currency volatility and constraining other actions and actors.^12^ The concept of a gold standard implies an anchoring of value to one material object (in this case a fixed quantity of gold) that appears to exist outside of context yet is actually deeply situated socially and economically.

In the scientific context of developing and adopting NAMs, the use of rodents in drug development and testing, for example, is often identified as the gold standard that needs to be replaced. Yet, to focus on redefining this gold standard is to presume that the gold standard exists in the singular, and that it can be clearly defined in an agreed way such that something (singular) could be substituted for it. These assumptions are largely sufficient for those seeking to shift biomedical research away from its reliance on animal experiments. However, animal-dependent models are probably not the gold standard (whatever is meant by that term) in the first place. The deficiencies of experimental animal models are well recognised, and indeed these deficiencies are part of what drives the movement toward NAMs, in the hope that they can address some of the challenges that have been well recognised in failed attempts to translate the results from laboratory animal studies to the human clinical context.^13–14^

The most rapid moves toward NAMs are happening in regulatory contexts where, as science and technology studies scholar Sheila Jasanoff explains, knowledge for regulatory policy is produced in institutional settings and under criteria of validity that differ from those in ‘basic’ or ‘research’ science.^15^ In many other contexts — for example, in pharmacological testing and many forms of biomedical research — such animal models currently remain the best, albeit imperfect, ‘tools for the job’.^16^ In many ways, animal models more closely resemble working standards, which require constant calibration that permits them to become as closely aligned as possible to the reference standards that are meaningful to that community.

The standards associated with animal research and its replacements are now extremely diverse. These include:

— standards for safety testing and drug licensing;
— quality assurance standards around animal models or tissue cultures;
— gold standard protocols for studying different diseases;
— national animal care and welfare guidelines;
— institutional researcher conduct guidelines; and
— international standards from journals and learned societies that are associated with publication and open science.

In these varied domains, there are likely to be different ideas of what the gold standards are (or should be). Some of these standards may align with others — for instance, the increasing emphasis on transparent reporting standards is closely related to standards about experimental replicability, publication practices and research integrity.^17^ However, there may also be divergent understandings related to standards — for example, about the continued use of oral gavage as the gold standard for drug delivery in drug development, which is now considered to be suboptimal based on animal welfare standards.^18^

Where differences and tensions exist, changing standards will require an understanding of the complexities associated with a range of diverse practices. This problem is further complicated by the range of heterogenous scientific practices that are typically grouped under the label of NAMs, all of which come with their own standards. These include stem cell-based assays,^19^ organoids and computer simulations, along with a range of other *in vitro*, *in silico*, or chemistry-based methods. Furthermore, NAMs are not always about *replacement*: the idea of ‘replacing the gold standard’ assumes that experimental animals (most likely rodents) will be replaced by an equivalent and even superior non-animal standard. However, many scientists view NAMs as adjuncts or components of experimental systems that are most useful when utilised in conjunction with animal-based, and sometimes human, research. Hence, NAMs are likely to be frequently integrated alongside animal models as a form of calibration toward the (usually) human reference standard.^20^

Considerable work will be required to develop and disseminate both the formal data and the practical know-how that are needed to shift practices toward NAMs, and to consider how evidence is produced and used with these new methods, and how risks and benefits (notably the benefits of not using animals as compared to the risks of not generating accurate information due to the use of less established methods) are understood in any one research domain and how it relates to others. Successful development and adoption of NAMs requires reflection on what is needed to co-ordinate evidence and embed practices across diverse standards for disease modelling, drug licensing, quality assurance, researcher conduct, funder requirements and publication ethics.

## Standards evolve over time and are enacted in different ways

Ideas of a gold standard that are based on experimental animals often mask the complications associated with such animals, which are simultaneously material entities and models.^21^ It is well recognised that the use of animal models assumes that another species, typically simpler in relevant ways, can be used as a so-called ‘model’ for more complex organisms, notably humans.^22^ What is less well recognised (particularly by scientists) are the contingencies, social and economic relationships and path dependencies that characterise the history of certain organisms (especially rodents) becoming the gold standard for biomedical and many other forms of research.^23–27^

Understanding how standards become established in the first place, as well as how they are sustained and change over time, are crucial initial steps for any processes intended to elicit intentional change (such as the greater use, or replacement, of animal models by NAMs). Importantly, processes of establishing and maintaining standards must address sociocultural issues as much as relevant scientific factors, because standards come into being and change through a range of processes that go well beyond scientific or technological requirements.^28^ For example, from the historian Robert Kohler’s account of how *Drosophila melanogaster* became a model organism, we learn that the ways in which the fruit fly was embedded in specific forms of human community and practices (e.g. via the ‘Fly Group’ and the ‘Fly Room’) were as important as its biology (including its small size, visible chromosomes and rapid reproductive cycles) in accounting for its success.^29^ More recently, geographer Bronwyn Parry has explored how the specific mouse strains provided by the Jackson Laboratory have functioned as a ‘gold standard’ that supports a global biotechnological commons of material and knowledge exchange.^30^

Norms that are understood as standards can travel far from their original intended context of use and prove to be remarkably persistent or deeply embedded into scientific practices once established, through notions ranging from experimental control to animal care and more. For instance, the historian Karen Rader argues that the genetically standardised mouse succeeded as a standard organism, even though the science for which it was created (mammalian genetics) initially did not, in part because of the limits of the available technologies.^23,31^ This demonstrates how the development of standardised organisms reflects changing social as well as disciplinary ‘ecologies of knowledge’.^29^ Rader cites the etymology of a ‘standard’, which includes older meanings such as ‘banner’ and ‘rallying point’, and shows how the use of a particular animal model can become entangled within institutional, disciplinary, and even personal, identities — for example, users of standardised mice were known as ‘mouse people’^31^ in the same way that ‘drosophilists’ were thought of as ‘fly people’.^29^ Hence, standards are always accompanied by a rich network of not only scientific and technological factors, but also sociocultural, historical, conceptual and other considerations that help to establish and maintain them. We can expect to see similar issues emerging around the different materials and technologies involved in NAMs.

Standards may also be reached through different modes of action. Animal research is known to involve both engineering standards and performance standards. Engineering standards are generally quantified outcome measures. These tend to be preferred by regulators, who require confirmed adherence to known standards, as in good laboratory practice (GLP). Engineering standards can also be used by centralised authorities seeking to move diverse countries or activities toward a common practice, as in the European Directive on animal research.^32^ Preferred in US approaches to animal care, performance standards are guidelines which identify a “desired outcome, [while providing] flexibility in achieving this outcome by granting discretion to those responsible”.^33^ Engineering and performance standards allocate authority, responsibility and expertise to different bodies, and there can be conflict between them as different scientific and national cultures meet in debates about harmonisation.^7^ International movements toward NAMs are likely to involve a similar mix of engineering standards (as in the adoption of new regulatory tests) and performance standards (as in the management of novel tissue cultures). Awareness of the cultural, as well as scientific, nature of standards — and the potential for conflict between them — is vital if new standards are to be implemented and adopted.

## Regulation and policy will be critical drivers, if structured appropriately

Scientific developments and technological innovations are often incapable of generating behavioural changes on their own. Hence, NAMs will not be used on a widespread basis, unless this use is coupled with other drivers. It has been argued that the replacement of animal models has been slow in development, due not only to ‘scientific inertia’ but also to conservatism in scientific practices, policy and existing regulatory standards.^34^ An illuminating historical example is that, despite having a clear scientific rationale supported by technological advances, mid-20th century animal researchers were slow to adopt purpose-bred standard laboratory animals for a number of reasons — not least of which was their economic cost, preferring to continue to use agricultural and domestic animal breeding networks to source their animals.^35^ Various approaches were deployed to elicit behaviour change amongst researchers, such as leveraging the extra-scientific persuasive power of national and international governmental and non-governmental bodies that established guidelines requiring the use of laboratory-bred animals.^36^

Clearly, NAMs are most likely to be rapidly adopted if they are encouraged or required by regulation. In some senses, NAMs are often not actually new or novel — instead, it is their use in a new setting, coupled with allowances in regulatory and accompanying decision-making processes, that permit them to serve as replacements for, or complements to, traditional animal testing. Thus, whilst there is increasing interest by regulatory authorities (notably in recent years by the US Food and Drug Administration) in the potential of NAMs, a key challenge remains — namely that although confidence in the reliability of NAMs is growing slowly, the level of confidence in such replacements remains varied across different sectors and jurisdictions.^37^

Although current regulations regarding the assessment of human health effects of industrial chemicals and pesticides in the USA, Canada and the EU include some flexibility to permit the use of NAMs, how this flexibility is interpreted and when it is invoked or employed varies considerably across product type and regulatory scheme.^38^ It is highly likely that these types of interpretative differences will remain part of the regulatory processes associated with NAMs, creating many of the same social and political drivers and inhibitors that occurred with the implementation of the Three Rs.^39^ Even the absence of regulation can serve as a driver — consider the recent increased use of embryonic and larval fish models as alternatives to vertebrate models in environmental toxicology. Such approaches are often counted as NAMs, and are even considered as not involving animals, as regulations in many jurisdictions do not include protection for the early developmental life stages in non-mammalian species.^40^ Further, although those working in chemical regulation appear to agree that fundamental reforms are needed, they cite the complexities associated with the sheer numbers, combinations and pervasiveness of industrial chemicals in products and in the environment as significant practical barriers to change.^41^

The perceptions of companies that test and register new chemicals will play a significant role in the uptake of NAMs approaches and influence the extent to which conservatism is likely. If companies perceive that there is a regulatory requirement for animal study data, then NAMs will not be used (even if such methods provide significant cost savings, along with other obvious benefits). Thus, regulatory agencies will not gain experience of, or confidence in, data obtained by using these approaches, and consequently there will be limited incentives to adopt or develop NAMs.^36,42^ Enhancing opportunities for discussion between regulatory authorities and registering companies would help facilitate the uptake and development of NAMs. Such discussion would also ensure that resources are not wasted on developing and carrying out testing that is later found to be incomplete or unacceptable. There is reported disparity between regulatory authorities in their willingness to meet with stakeholders in advance of submissions for consideration for regulatory approval, but this type of engagement would clearly save time and resources, as well as encouraging knowledge sharing on both sides.^37^

A further inhibitor to the adoption of NAMs by companies with globally distributed product target markets, is the extent of international harmonisation of regulatory testing guidelines. Organisations such as the Organisation for Economic Co-operation and Development (OECD) have developed mechanisms aimed at reducing the duplication of testing, as well as harmonising test guidelines across member countries. However, modernisation of regulatory approaches is needed to maximise opportunities for the timely uptake of NAMs. For example, a key stumbling block has been the reliance of regulatory authorities on ‘predictive capacity’ for determining acceptance of a test — a norm that is usually determined by comparison of the new results with those obtained by using traditional animal testing methods (reinforcing ideas of a ‘gold standard’). NAMs will fail this type of test when they do not provide the same information as animal models, even though this information may be more relevant to human health and provide greater mechanistic insights.^43^

An additional complication is that legislation governing animal research ethics is often local to the jurisdiction where the research occurs, particularly in the case of basic research or preclinical drug development. There is limited consistency between jurisdictions in the format or operation of these laws, despite a few unifying features, such as a focus on the Three Rs, and the requirements for harm–benefit assessments by an ethics review committee comprising certain types of members (e.g. biological researchers, ethicists, community representatives, and so on).^43^ Ethics committees often lack biostatisticians who can analyse complex study designs, as this role type is not typically mandated.^44^ The assessment of compliance with mandates on replacement (or even reduction) requires that investigators engage in due diligence to determine that valid alternatives do not exist. This process equally requires that ethics committee members have the requisite knowledge (or access to the relevant research literature) and the necessary skills to assess such claims.^45^ This requirement is additionally challenging because research protocols are often based in disciplines separate from the emerging, and often interdisciplinary, fields where alternatives are being developed, such as the computer sciences, stem cell research, or biotechnology, as was recognised long ago by the philosopher Denise Russell.^46^

These problems are not insurmountable, if there is improved access to training in NAMs and the fostering of more collaborative, transdisciplinary research networks. However, significant structural changes and investment will be required for these to develop, as well as shifts in how key standards associated with scientific methodology, such as replicability and predictability, are understood. Animal research legislation is generally silent on methods for fostering these practices, for ensuring that they are occurring where appropriate or, more generally, for creating positive duties for institutions to undertake with regard to alternatives. Hence, the regulatory process, in many jurisdictions, is failing at the NAMs implementation stage, which includes the failure to establish the necessary standards required.

## The need to articulate shared social and ethical norms

For those individuals and groups who have enthusiastically endorsed NAMs and are critical of animal-based research, NAMs may appear to offer a way to circumvent the ethical issues that arise in animal research. Most obviously, if animals are not involved in research, then they are not subject to the harms associated with such research. In addition, those who work in animal research (such as research scientists, animal care workers, laboratory technicians, etc.) avoid the potential harms which may accompany this work, including physical, physiological and moral injuries.^47,48^

However, NAMs will not ‘solve’ all of the ethical issues that are associated with biomedical research. For instance, their uptake will not diminish the need to articulate justifications for the questions pursued or for reflection on the outcomes that may result, including whether and how research is aligned with public benefit and social needs, and what the risks are as compared to the benefits. NAMs are not ethically neutral. In fact, they generate their own ethical issues, albeit ones that are different from those typically raised by animal research. For example:

— methods such as data mining can raise privacy worries, and it also is not clear that data produced for one purpose will be valid for another;
— environmental concerns may be associated with the use of vast computing resources to run simulations;
— organoids and stem cell cultures are often accompanied by an array of challenges, particularly related to informed consent; and
— the increased use of humans in lieu of experimental non-human organisms may place pressures on the most vulnerable members of society, who are more likely to be targeted for recruitment for biomedical research.

Any moves away from animal models as the dominant way of addressing research questions in biomedicine will necessarily involve a shift in power relations and invoke ethical concerns. For instance, during the period of transition to NAMs, already vulnerable animal technicians and carers may be exposed to the stresses of job precarity, as well as increased stigmatisation — given that in the face of apparently preferable alternative methods, animal-based work may be increasingly regarded as unpalatable or even immoral.

NAMs present clear opportunities for ethical and social norms to play crucial roles in shaping their development and uptake. Consideration of these norms by researchers and regulators is important, as it can help to establish and maintain the social licence that is central for good and robust scientific and medical research.^37,49^ It will be essential to be as transparent as possible about the assessment processes associated with various types of NAMs, including their inherent uncertainties and limitations. Intra- and inter-agency collaborations also have been argued to be important in strengthening scientific, regulatory and public confidence in NAMs.^37^ Such processes can be supported by:

— establishing independent review processes for each type of NAM and its intended application;
— reviewing the associated standards, once norms begin to be articulated;
— considering the use of registries and other open communication networks; and
— publishing negative or null results and eliminating various other sources of publication bias.

It also will be essential to revisit norms associated with research integrity, including establishing standards for peer review related to the use of appropriate methodologies and the experimental outcomes, reproducibility, and so on, rather than simply transferring existing norms from animal experimentation to NAMs. These existing norms are likely to be outdated and inappropriate for use in their new context.

## Conclusions

The complexities associated with standards discussed in this paper through reflection on humanities and social science research about animal experimentation, indicate why trying to establish NAMs as the ‘gold standard’ requires consideration of the nature of standards and their diversity, and the practices associated with standards. Any conversations about changing from one gold standard approach in science to another must be attentive to the kinds of standards being discussed, and the problems associated with the concept of a ‘gold standard’. As noted by the science and technology studies scholar Annamaria Carusi, “The age of the ‘gold standard’ of animal tests seems to have passed, but it has not yet been definitively replaced”.^40^ Efforts to promote NAMs should always be accompanied by reflection on the rich history of practices in the accompanying fields of research, the underlying concepts and norms that ground those practices, and the geography that situates past and current practices. An understanding of all these factors is required to begin to envision how change may be able to happen, including the potential drivers of, and barriers to, such change.

Additional research in the humanities and social sciences is needed, particularly on the following topics:

— analysis of drivers of and impediments to change in scientific practices associated with NAMs, including disciplinary, social and cultural norms, which are likely to be simultaneously global and local, and hence riddled with tensions;
— exploration of institutional structures and visions for their roles in change in the context of NAMs: for instance, universities will be as important as companies, particularly as the former provide training and instil norms for practising scientists;
— investigation of which types of research trajectories are more readily (or likely to be) able to accommodate NAMs and thus transition to their use (and which are not). For instance, academic and basic researchers working on immune system responses or the brain, tend to emphasise the need to study the whole intact animal, whereas clearly toxicity testing is already shifting to new approaches;
— articulation of the values and norms that should be associated with training the next generation of researchers and laboratory and animal technicians, particularly during periods of transition, or in domains where NAMs are likely to be used together with animals; and
— exploring debates about what should lead change in this domain. For instance, various advocates for NAMs focus on standards for scientific evidence, institutional governance, regulation and law, investment or economics, or social values, among other factors.

However, what may be a highly efficacious pressure point from one point of view might be in tension with, or incompatible with, other approaches to fostering change to promote use of NAMs.

Debate and the explicit establishment of standards must be key aspects of the processes associated with the development and implementation of NAMs, although what is deemed ‘best practice’ is likely to shift over time and vary across different types of research, jurisdictions and other sociocultural contexts. We must be cautious about how we think about NAMs as a new ‘gold standard’, including their potential advantages and likely shortcomings. It is critical to establish shared and transparent norms well before NAMs become entrenched simply by accretion or due to technological innovations.

## References

[1] Davies G Greenhough B Hobson-West P, et al. (eds) Researching animal research: What the humanities and social sciences can contribute to laboratory animal science and welfare. Manchester: University of Manchester Press, 2024, 480 pp.

[2] Grimm H Biller-Andorno N Thorsten B , et al. Advancing the 3Rs: Innovation, implementation, ethics and society. Front Vet Sci 2023: 10. 10.3389/fvets.2023.1185706 PMC10310538 37396988

[3] Creager ANH Lunbeck E Wise MN (eds). Science without laws: Model systems, cases, exemplary narratives. Chapel Hill, NC: Duke University Press, 2007, 287 pp.

[4] Lampland M Star SL (eds). Standards and their stories: How quantifying, classifying, and formalizing practices shape everyday life. Ithaca, NY: Cornell University Press, 2008, 264 pp.

[5] Barry A . Technological zones. Eur J Soc Theory 2006; 9: 239–253.

[6] Vasbinder MA Locke P . Introduction: Global laws, regulations, and standards for animals in research. ILAR J 2016; 57: 261–265. 29117408 10.1093/ilar/ilw039

[7] Davies G . Locating the ‘culture wars’ in laboratory animal research: National constitutions and global competition. Stud Hist Philos Sci 2021; 89: 177–187. 34455260 10.1016/j.shpsa.2021.08.010 PMC8513693

[8] Bolden J Smith K . Application of recombinant factor C reagent for the detection of bacterial endotoxins in pharmaceutical products. PDA J Pharm Sci Technol 2017; 71: 405–412. 28733334 10.5731/pdajpst.2017.007849

[9] Gorman R. Atlantic horseshoe crabs and endotoxin testing: Perspectives on alternatives, sustainable methods, and the 3Rs (Replacement, Reduction, and Refinement). Front Mar Sci 2020: 7. 10.3389/fmars.2020.582132 PMC7612741 35591980

[10] Gorman R. Outside of regulations, outside of imaginations: Why is it challenging to care about horseshoe crabs? In: Davies G Greenhough B Hobson-West P , et al. (eds) Researching animal research: What the humanities and social sciences can contribute to laboratory animal science and welfare. Manchester: University of Manchester Press, 2024, pp. 57–79.

[11] Baker E Ponder J Oberdorfer J , et al. Barriers to the use of recombinant bacterial endotoxins test methods in parenteral drug, vaccine and device safety testing. Altern Lab Anim 2023; 51: 401–410. 37855095 10.1177/02611929231204782

[12] Eichengreen B . Globalizing capital: A history of the international monetary system. 3rd ed.. Princeton, NJ: Princeton University Press, 2017.

[13] Hartung T . Toxicology for the twenty-first century. Nature 2009; 460: 208–212. 19587762 10.1038/460208a

[14] Leenaars CHC Kouwenaar C Stafleu FR , et al. Animal to human translation: A systematic scoping review of reported concordance rates. J Transl Med 2019; 17: 22. 31307492 10.1186/s12967-019-1976-2 PMC6631915

[15] Jasanoff S . The fifth branch: Science advisers as policymakers. Cambridge, MA: Harvard University Press, 1990, 318 pp.

[16] Clarke AE (ed). The right tools for the job: At work in the twentieth-century life sciences. Princeton, NJ: Princeton University Press, 1992, 378 pp.

[17] Percie du Sert N Hurst V Ahluwalia A , et al. The ARRIVE guidelines 2.0: Updated guidelines for reporting animal research. J Cereb Blood Flow Metab 2020; 40: 1769–1777. 32663096 10.1177/0271678X20943823 PMC7430098

[18] Nilsson FAM Jirkof PD Johanson P , et al. Systematic review oral gavage alternatives for mice and rats. Internet Archive*,* https://archive.org/details/osf-registrations-4qtwd-v1 (2023, accessed 8 May 2024).

[19] Thompson C . On the research subject and the animal model. In: Good science: The ethical choreography of stem cell research. Cambridge, MA: The MIT Press, 2013, pp. 189–222.

[20] Krewski D Acosta Jr. D Andersen M , et al. Toxicity testing in the 21st century: A vision and a strategy. J. Toxicol. Environ Health B Crit. Rev 2010; 13: 51–138. 20574894 10.1080/10937404.2010.483176 PMC4410863

[21] Ankeny RA Leonelli S . Model organisms. Cambridge: Cambridge University Press, 2020, 90 pp.

[22] Ankeny RA Leonelli S . What’s so special about model organisms? Stud Hist Philos Sci 2011; 42: 313–323.

[23] Rader KA . Making mice: Standardizing animals for American biomedical research, 1900–1955. Princeton, NJ: Princeton University Press, 2006, 312 pp.

[24] Davies G . Arguably big biology: Sociology, spatiality and the knockout mouse project. Biosocieties 2013; 8: 417–431.

[25] Huber L Keuck LK . Mutant mice: Experimental organisms as materialised models in biomedicine. Stud Hist Philos Sci 2013; 44: 385–391. 10.1016/j.shpsc.2013.03.001 23545252

[26] Ankeny RA Leonelli S Nelson NC, et al. Making organisms model humans: Situated models in alcohol research. Sci Context 2014; 27: 485–509. 25233743 10.1017/s0269889714000155 PMC4274764

[27] Nelson NC . Model behavior: Animal experiments, complexity, and the genetics of psychiatric disorders. Chicago, IL: University of Chicago Press, 2018, 262 pp.

[28] Dietrich MR Ankeny RA Crowe N , et al. How to choose your research organism. Stud Hist Philos Biol Biomed Sci 2020; 80: 101227. 31883711 10.1016/j.shpsc.2019.101227

[29] Kohler R . Lords of the fly: Drosophila genetics and the experimental life. Chicago, IL: University of Chicago Press, 1994, 344 pp.

[30] Parry B . Patents and the challenge of ‘open source’ in an emergent biological commons or … the strange case of Betty Crocker and the mouse. Biosocieties 2020; 15: 294–315.

[31] Rader KA. ‘The mouse people’: Murine genetics work at the Bussey Institution, 1909–1936. J Hist Biol 1998; 31: 327–354. 11623531 10.1023/a:1004389408064

[32] Olsson IAS da Silva SP Townend D , et al. Protecting animals and enabling research in the European Union: An overview of development and implementation of directive 2010/63/EU. ILAR J 2016; 57: 347–357. 29117403 10.1093/ilar/ilw029

[33] National Research Council . Guide for the care and use of laboratory animals. Washington, DC: National Academies Press, https://www.ncbi.nlm.nih.gov/books/NBK54054/ (2011, accessed 8 May 2024).

[34] Lohse S. Scientific inertia in animal-based research in biomedicine. Stud Hist Philos Sci 2021; 89: 41–51. 34333156 10.1016/j.shpsa.2021.06.016

[35] Kirk RGW. ‘Wanted — standard guinea pigs’: Standardisation and the experimental animal market in Britain ca. 1919–1947. Stud Hist Philos Biol Biomed Sci 2008; 39: 280–291. 18761280 10.1016/j.shpsc.2008.06.002 PMC3302564

[36] Kirk RGW . A brave new animal for a brave new world: The British Laboratory Animals Bureau and the Constitution of International Standards of Laboratory Animal Production and Use, circa 1947–1968. Isis 2010; 101: 62–94. 20575490 10.1086/652689 PMC3299560

[37] Sewell F Alexander-White C Brescia S , et al. New approach methodologies (NAMs): Identifying and overcoming hurdles to accelerated adoption. Toxicol Res 2024; 13: tfae044. 10.1093/toxres/tfae044 PMC10964841 38533179

[38] Stucki AO Barton-Maclaren TS Bhuller Y , et al. Use of new approach methodologies (NAMs) to meet regulatory requirements for the assessment of industrial chemicals and pesticides for effects on human health. Front Tox 2002; 4. 10.3389/ftox.2022.964553 PMC9475191 36119357

[39] Schiffelers MJ Blaauboer BJ Fentener van Vlissingen JM , et al. Factors stimulating or obstructing the implementation of the 3Rs in the regulatory process. ALTEX 2007; 24: 271–278. 18288426 10.14573/altex.2007.4.271

[40] Halder M Leonard M Iguchi T , et al. Regulatory aspects on the use of fish embryos in environmental toxicology. Integr Environ Assess Manag 2010; 6: 484–491. 20821708 10.1002/ieam.48

[41] Carusi A . Chemicals regulation and non-animal methods: Displacing the gold standard. Wellcome Open Science 2024, https://wellcomeopenresearch.org/articles/9-167 (accessed 13 May 2024). 10.12688/wellcomeopenres.20581.2 PMC11391182 39267989

[42] Swaters D van Veen A van Meurs W , et al. A history of regulatory animal testing: What can we learn? Altern Lab Anim 2022; 50: 322–329. 35983829 10.1177/02611929221118001

[43] van der Zalm AJ Barroso J Browne P , et al. A framework for establishing scientific confidence in new approach methodologies. Arch Toxicol 2022; 96: 2865–2879. 35987941 10.1007/s00204-022-03365-4 PMC9525335

[44] Brønstad A Newcomer CE Decelle T , et al. Current concepts of harm–benefit analysis of animal experiments: Report from the AALAS–FELASA Working Group on harm–benefit analysis — Part 1. Lab Anim 2016; 50: 1–20. 27188275 10.1177/0023677216642398 PMC5815836

[45] Ankeny RA Whittaker AL Ryan M , et al. The power of effective study design in animal experimentation: Exploring the statistical and ethical implications of asking multiple questions of a data set. Brain Behav Immun 2023; 112: 163–172. 37315700 10.1016/j.bbi.2023.06.012

[46] Russell D. Why animal ethics committees don’t work. Between the Species 2012; 15: 127–142.

[47] Akhtar A. The flaws and human harms of animal experimentation. Camb Q Healthc Ethics 2015; 24: 407–419. 26364776 10.1017/S0963180115000079 PMC4594046

[48] Johnson J Smajdor A . Human wrongs in animal research: A focus on moral injury and reification. In: Herrmann K Jayne K (eds) The ethics of animal experimentation: Working towards a paradigm change. Leiden: Brill Publishing, 2019, pp. 305–317.

[49] Raman S Mohr A . A social licence for science: Capturing the public or co-constructing research? Soc Epistemol 2014; 28: 258–276.

